# Clinically Relevant Outcome Measures in Women With Adrenoleukodystrophy

**DOI:** 10.1002/acn3.70314

**Published:** 2026-01-20

**Authors:** Chenwei Yan, Elizabeth I. Pierpont, Amena S. Fine, Reena V. Kartha

**Affiliations:** ^1^ Department of Experimental and Clinical Pharmacology, College of Pharmacy Center for Orphan Drug Research, University of Minnesota Twin Cities Minneapolis Minnesota USA; ^2^ Division of Clinical Behavioral Neuroscience, Medical School University of Minnesota Twin Cities Minneapolis Minnesota USA; ^3^ Department of Neurogenetics Moser Center for Leukodystrophies, Kennedy Krieger Institute Baltimore Maryland USA; ^4^ Department of Neurology Johns Hopkins University School of Medicine Baltimore Maryland USA

**Keywords:** adrenoleukodystrophy, clinical outcomes, myeloneuropathy, women

## Abstract

Adrenoleukodystrophy is a rare inherited peroxisomal disease caused by pathogenic variants in the *ABCD1* gene located on the X chromosome. Although the most severe central nervous system and adrenal complications typically affect only men with adrenoleukodystrophy, the majority of women develop myeloneuropathy symptoms in adulthood. In observational studies of women with adrenoleukodystrophy, several clinical rating scales have been used to assess disease manifestations and capture differences between asymptomatic and symptomatic women. To facilitate development of treatments to address symptoms in women, there is a need to identify clinical outcome measures that can sensitively assess disease progression and treatment responses. The goals of this scoping review were to: (1) identify and review clinically relevant assessment scales that have been utilized to capture disease manifestations in women with adrenoleukodystrophy, and (2) provide recommendations on key objectives for further research.

## 1 Introduction

Adrenoleukodystrophy (ALD) is a rare, progressive X‐linked metabolic disorder with an estimated incidence of 1 in 16,800 to 21,000 births across all ethnic groups [1]. ALD is caused by pathogenic variants in the *ABCD1* gene located on the X chromosome. In terms of pathophysiology, the presence of a defective peroxisomal transporter leads to abnormal accumulation of very long‐chain fatty acids within cells, which can damage the adrenal cortex, spinal cord, and brain [1]. The clinical progression of ALD varies widely and the main phenotypes observed in individuals with ALD and their management are summarized in (Table 1) [1, 2, 3].

**TABLE 1:** Clinical phenotypes in adrenoleukodystrophy.

Phenotypes	Prevalence [1, 2]	Diagnostics/Symptoms [1, 2]	Treatment options
*Men*			
Childhood cerebral ALD	~35%	Rapidly progressive inflammation and damage to brain; progressive behavioral, cognitive, and neurologic deficits; testicular dysfunction	Hematopoietic stem cell transplant (HSCT) can halt disease progression if provided at an early stage Gene therapy SKYSONA (FDA approved in 2023) for indicated individuals
Adult cerebral ALD	~20%–30%	Parallel to childhood cerebral ALD with a later onset; may present with marked psychiatric disturbance	HSCT if identified early
Adrenomyelo‐neuropathy (AMN)	Observed in most adult males with ALD, with onset in late 20s to middle age	Weakness, spasticity, pain, bladder and bowel dysfunction, impaired movement. Adrenal insufficiency may also be present	Symptom management
Addison's disease only	< 10%, since most continue on to develop AMN	Adrenal insufficiency	Corticosteroid replacement therapy
*Women*			
Myelo‐neuropathy	~20% under age 40; ~80% by age 50	Similar to AMN in males except onset is typically later. Adrenal involvement is extremely rare	Symptom management
Cerebral demyelinating disease	Rare		
Adrenal insufficiency	Rare		

As an X‐linked disorder, ALD was regarded as a disease affecting only men for many years, while women were referred to as “carriers.” As a result, limited early research focused on women [3, 4]. Nevertheless, the diagnosis of ALD and the development of neurological symptoms can place a significant burden on women's personal health, functioning, reproductive decisions, and family life. Some women come to seek medical care when they are asymptomatic because of their family history. Others already have neurological involvement such as ataxia, spasticity, weakness, neurogenic bowel and bladder symptoms, and neuropathic pain or sensory changes [2]. The neurological symptoms in women tend to develop in middle age and progress more slowly compared to men. Motor impairment is positively correlated with age [3]. Bladder and/or bowel dysfunction can impact younger age groups of women with ALD. There is currently no definitive therapy; hence, symptom management is primarily used for women. To enable effective monitoring of potential therapies, sensitive, accurate outcome measures are needed to assess progression and treatment response in women. This review aims to consolidate the limited literature on the clinical outcomes assessments (COAs) that have been used in research involving women with ALD, informing the design of future clinical trials.

## 2 Methods

A literature search was performed to identify studies relevant to adult women affected by ALD, using methods consistent with the guideline provided in the Preferred Reporting Items for Systematic Reviews and Meta‐Analyses (PRISMA) extension for scoping review checklist. Peer‐reviewed, observational studies that included measurement of clinical symptoms among women affected by ALD were included. Review papers, clinical management guidelines, and case reports were excluded.

**FIGURE 1:**
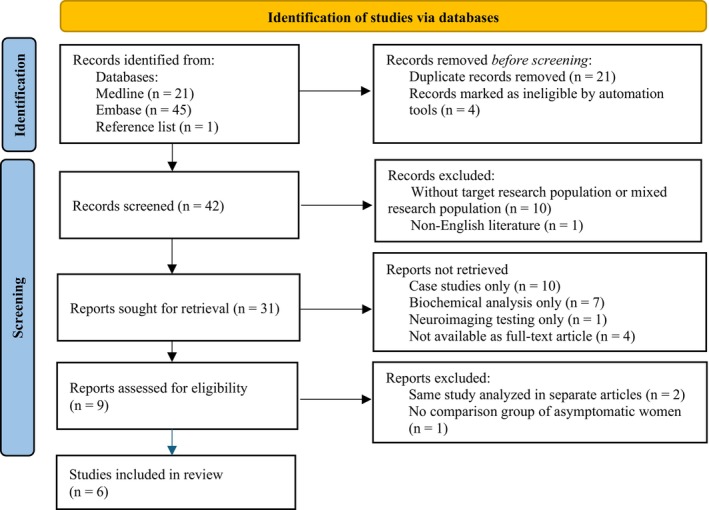
Study selection process illustrated by the PRISMA (Preferred Reporting Items for Systematic Reviews and Meta‐Analyses) flow diagram.

Figure 1 shows the study screening process. A literature search was conducted in 2 databases: Medline (via PubMed) and Embase (via Ovid) up until December 2024. The search terms were developed by combining the 2 key characteristics of interest: belonging to a population of adult women and being diagnosed with ALD. Only English‐language studies that were published in peer‐reviewed journals and available as full‐text articles were included. A single reviewer screened both titles and abstracts. To ensure that the search was comprehensive, reference lists from retrieved, eligible articles were also manually searched.

## 3 Results

### 3.1 Results of Literature Search

The initial search identified 21 articles from Medline and 45 articles from Embase. A preliminary screening process was conducted to remove duplicates and those with unrelated titles or abstracts. Next, full‐text screening was performed to retrieve the studies that met the inclusion criteria. The search resulted in 6 articles, summarized in Table S1 with respect to their study design and population selection [4, 5, 6, 7, 8, 9]. All published studies focused on adult women (> 18 years of age), further defined into asymptomatic and symptomatic groups. Five studies used the presence of myelopathy or neuropathy to differentiate symptomatic patients [4, 5, 6, 8, 9], whereas one study relied on neurological examination and a clinical symptom scale [7].

### 3.2 Clinical Outcomes Assessments

The 6 studies meeting the inclusion criteria all used either clinician‐completed or patient‐reported COAs to capture disease progression and its impact on the study patients. While myeloneuropathy is the major phenotype observed in affected women, a few studies also assessed mental health and physical disability observed in women (Table 2).

**TABLE 2:** Clinical assessments included in observational studies of women with ALD.

Outcome measured	Assessment instrument	Rater
Neurological impairment	Adult ALD Clinical Symptom Scale (AACS) [7]	Clinician
Disability status and motor function	Academic Medical Center Linear Disability Scale (ALDS) [4, 8] Expanded Disability Status Scale (EDSS) [8]	Clinician
Sensory‐motor disorder	International Restless Legs Syndrome Study Group rating scale (IRLS) [9]	Patient
Myelopathy	Japanese Orthopedic Association (JOA) Score [5, 6] Severity Score System for Progressive Myelopathy (SSPROM) [5, 6]	Clinician
Health‐related quality of life	Short Form (36) Health Survey (SF‐36) [8, 9]	Patient
Mental health or physical health symptoms	Beck Depression Inventory (BDI) [9] Beck Scale for Suicide Ideation (BSSI) [9] Brief Pain Inventory—short form (BPI‐sf) [9] Modified Fatigue Impact Scale (MFIS) [9] Pittsburgh Sleep Quality Index (PSQI) [9] Female Sexual Function Index (FSFI) [9]	Patient

### 3.3 Neurological Impairment

#### 3.3.1 Adult ALD Clinical Symptom Scale

The AACS is a disease‐specific COA to evaluate neurological impairment in adults with ALD. This 10‐item questionnaire covers motor, bladder, sensory, and cerebral functions and the score ranges from 0 to 24, with higher scores indicating severe neurological impairment [10]. One study reported longitudinal AACS data from a cohort of women (*n* = 32) with confirmed *ABCD1* gene variants and three recorded visits (baseline and 2 follow‐up visits) in a 7‐year period [7]. They observed that at baseline, age was the most significant predictor of ALD progression (odds ratio [OR] 1.21, *p* < 0.05, 95% confidence interval [CI] 1.03–1.42), with the rate of disease progression quantified as 0.24 AACS points per year after the third or fourth decade of life [7]. Note that the AACS captures the clinician's impression of disease severity based on history obtained during patient interview; thus, it is unclear to what extent this instrument captures symptoms and changes that are clinically meaningful to the patient. Further, a notable limitation in applying the AACS with female cohorts is that it contains a ‘cerebral function’ domain that is unlikely to be relevant due to the very low risk of cerebral involvement. As such, points assigned in this domain are likely to reflect secondary psychosocial burden of the disease (e.g., stress from experiences of loss, coping with disease burden) rather than primary neurological symptoms that could be responsive to disease‐modifying therapies [1, 2, 9].

### 3.4 Disability Status and Motor Function

#### 3.4.1 Academic Medical Center Linear Disability Scale (ALDS)

The ALDS is a validated clinical tool to measure physical disability in patients with chronic diseases [11]. Items focus on disabilities affecting daily activities, with a lower ALDS score indicating higher disability. A cross‐sectional study involving a cohort of 46 women with ALD in the Netherlands [4] found that symptomatic women had significantly lower ALDS scores than asymptomatic women (*p* < 0.05) [4]. Further, comparison of these observations to a prospective cohort study in Parkinson's disease [12] revealed that symptomatic women with ALD have levels of disability comparable to patients with Parkinson's disease 1 year after diagnosis (*p* < 0.01) [4]. The ALDS has also been utilized to capture spinal cord disease progression. Huffnagel and colleagues obtained follow‐up data on 34 of 46 (74%) women enrolled in the previously mentioned study and 19 newly identified women; median time between baseline and follow‐up was 7.8 years [8]. At baseline, there was again a difference in ALDS scores between symptomatic and asymptomatic women (*p* < 0.0005). Higher age was associated with a lower ALDS score and thus greater disability (*p* = 0.045) [8]. Moreover, asymptomatic women had a significantly higher ALDS score (0.68 points) than women who had symptoms for more than 10 years (*p* = 0.019) [8]. No significant change was observed in ALDS scores after a follow‐up of approximately 8 years.

#### 3.4.2 Expanded Disability Status Scale (EDSS)

The EDSS was originally developed as a standardized measurement in multiple sclerosis clinical trials and assessment. Scores range from 0 to 10, with a higher EDSS score indicating greater disability [13]. Similar to the ALDS, baseline differences in EDSS scores were found between symptomatic and asymptomatic women in the Netherlands cohort mentioned above (*p* < 0.0005). Additionally, asymptomatic women had a consistently lower EDSS score than women who had symptoms for more than 10 years (*p* < 0.0005) [8]. However, when including only women with both baseline and follow‐up assessments (*n* = 34), the EDSS showed significant longitudinal change (*p* < 0.0005) whereas the ALDS did not [8]. Given its greater sensitivity, the EDSS may be of greater utility than the ALDS to show natural history progression. Nevertheless, the EDSS is recognized to have limitations as a clinical trial endpoint in this population because of the large cohort size and a lengthy study period (e.g., 8 years) required to capture the slowly progressive changes that occur.

### 3.5 Sensory‐Motor Disorder

#### 3.5.1 International Restless Legs Syndrome Study Group Rating Scale (IRLS)

Restless Legs Syndrome (RLS) is characterized by an urge to move the legs, especially during rest, and can contribute to daily motor difficulties. The IRLS was initially used by Schäfer and colleagues to assess the presence and severity of RLS in women with ALD, who highlighted that symptomatic women with ALD had higher total IRLS scores than asymptomatic women (*p* < 0.001), which indicates more severe RLS [9]. Additionally, a pilot study combined diagnostic interviews, retrospective chart review, and functional gait assessment to determine RLS prevalence in adults with ALD (10 women and 3 men included) [14]. RLS was found to occur in 40% of participants, with women more commonly affected than men; those with RLS showed more signs and symptoms of myelopathy.

### 3.6 Myelopathy

#### 3.6.1 Japanese Orthopedic Association (JOA) Score and Severity Score System for Progressive Myelopathy (SSPROM)

The JOA score and SSPROM are both myelopathy scales. The JOA score has been validated for use with degenerative vertebral diseases evaluating motor function, sensory, and bladder function. The SSPROM, similar to the JOA, was developed to address changes in progressive myelopathies covering motor disability, sphincter dysfunction, spasticity, and sensory losses [15]. Lower scores on both scales indicate worsening symptoms. Both were used by Habekost and colleagues in their baseline study (*n* = 33) and a follow‐up study with the same cohort [5, 6].

In their initial study, when comparing symptomatic women to asymptomatic women, they found that the symptomatic group was significantly older and had lower JOA and SSPROM scores (*p* < 0.05), indicating greater disability [5, 6]. The duration of disease also correlated with both JOA and SSPROM. It is worth noting that results from neurophysiological testing, which included nerve conduction studies and somatosensory evoked responses, suggested that neurological impairment was better evaluated by these clinical scales than by neurophysiology. Both JOA and SSPROM scales were able to discriminate symptomatic from asymptomatic women, while no substantial alterations on peripheral neurophysiology were found.

Symptomatic women in this study were invited to participate in follow‐up visits with the goal of characterizing trajectories of progression on these scales. There were 21 follow‐up evaluations of the JOA and SSPROM at a mean of 9 months [6]. 14 individuals were evaluated once, 9 were evaluated twice, and 6 were evaluated three times. The authors estimated that the JOA score at disease onset was 16.6 points, with an average annual decrease of 0.42 points (*p* < 0.001). The estimated mean value of the SSPROM score at the disease onset was 96.5 points with an average annual decrease of 1.87 points (*p* < 0.001) [6]. While these results suggest that change over time was captured, the clinical relevance of these levels of decrease needs‐ further study, along with examination of associations with other biomarkers of disease severity.

### 3.7 Quality of Life and Mental Health

The SF‐36 is a widely used, easily accessible self‐report questionnaire that measures health‐related quality of life in both physical and mental health domains [16]. In an early study, SF‐36 scores in women with ALD were not significantly different between asymptomatic and symptomatic women in either physical or mental components, although a trend suggesting greater physical health burden was observed in those who were symptomatic [4]. Specific subdomains of SF‐36 were further analyzed with additional data and results showed that between symptomatic and asymptomatic women, there was a statistical difference in SF‐36 physical functioning (*p* = 0.005) and overall general health (*p* = 0.004) at baseline [8]. However, similar to ALDS score observed in this cohort, longitudinal assessment of women at baseline and follow‐up did not show significant differences in SF‐36 [8].

A more recent quality of life study with a much larger female cohort (*n* = 180) had different findings [4, 9]. Participants received the SF‐36 via a patient advocacy organization (European Leukodystrophies Association International). Results highlighted that symptomatic women reported significantly decreased health‐related quality of life on all subscales of the SF‐36 compared to asymptomatic women (*p* < 0.05). The groups were most similar in mental health domains and most discrepant in physical health quality of life [9]. Overall, the SF‐36 seems to be a useful tool for symptomatic women, providing gradation especially regarding physical health quality of life. Apart from SF‐36, this large cohort study also assessed a broad range of physical and mental health outcomes using self‐report rating scales, providing evidence that symptomatic women experienced greater symptoms of depression, fatigue, sleep disruption, sexual dysfunction, and pain interference. A summary of key observations from these measures is presented in Table 3 [9].

**TABLE 3:** Patient‐reported physical and mental health outcomes by symptom status in women with ALD.

Clinical scales	Results
Depression: BDI	Higher BDI scores (more severe depression) were reported in symptomatic as compared to asymptomatic study participants (*p* < 0.001)
Suicidal ideation: BSSI	There was no difference in BSSI scores (suicidal ideation) reported in symptomatic as compared to asymptomatic study participants (*p* = 0.102)
Pain: BPI‐SF	Compared to asymptomatic study participants, symptomatic women experienced more frequent pain (*p* < 0.001) and worse pain interference (*p* < 0.001)
Fatigue: MFIS	Higher MFIS scores (greater fatigue) were reported in symptomatic as compared to asymptomatic study participants (*p* < 0.001)
Sleep: PSQI	Higher PSQI scores (poorer sleep) were reported in symptomatic as compared to asymptomatic study participants (*p* < 0.001)
Sexual Dysfunction: FSFI	Compared to asymptomatic study participants, symptomatic women had lower FSFI scores (*p* < 0.001) and were more often classified as at risk of sexual dysfunction (*p* = 0.007)

*Note*: The indicated *p*‐values were derived using *t*‐tests (continuous variables) and *χ* ^²^ tests (categorical variables) [9].

## 4 Discussion

In recent years, there has been increased recognition of neurological and neuropsychological symptoms experienced by women with ALD. This scoping review aimed to provide an overview of COAs that have been used in observational studies to study the differential experiences of asymptomatic and symptomatic women and to characterize disease progression over time. An ideal COA is anticipated to capture changes in physical, neurological, or social functions in response to disease progression and capture change within a feasible monitoring window. Among scales measuring physical disabilities, the EDSS but not the ALDS demonstrated the potential to measure progression in spinal cord disease but was only sensitive within a longer timespan (e.g., 8 years) that may be impractical for some trials. Similarly, JOA scores and SSPROM were able to discriminate symptomatic from asymptomatic women with a slow rate of neurologic progression. A key question needing further evaluation is whether the observed change in the scores correlates with other clinical or biological markers of disease progression. Finally, results suggest that quality of life measures, such as SF‐36 and other self‐report questionnaires, may be able to detect differences in certain subdomains of physical and mental health when administered within large patient cohorts.

### 4.1 Key Objectives for Future Research

From reviewing COAs reported in the literature and identifying gaps in research for women with ALD, we offer our perspective on priorities for future studies below.

#### 4.1.1 Focus on Relevant Concerns That Matter to Patients

A 2022 Patient‐Focused Drug Development (PFDD) meeting identified balance issues, incontinence, altered gait, and spasticity as top concerns for adults with ALD [17, 18]. Both men and women with ALD contributed to this meeting report. Future studies need to prioritize COAs that capture these patient‐centered symptoms. For example, interventions addressing mobility issues in women require measures that quantify stability or improvement in physical disability to accurately evaluate therapeutic responses.

#### 4.1.2 Determine the Time Scale Over Which Each COA Is Appropriate

Natural history studies should utilize longitudinal designs to identify the time scale over which each COA may be appropriately applied. Functional outcomes such as EDSS and SSPROM have also been evaluated in men with ALD to track disease progression. However, similar to women, the observed changes were small even in symptomatic men [19]. When using these scales, a longer follow‐up period beyond 5 years is desired, although this timeframe may not be feasible for most clinical trial designs. To address this issue of slow progression of neurological and functional change, studies may also choose to focus on alternative outcome measures in women with ALD. RLS has recently been linked with ALD and one study noted that 47.3% of women with ALD self‐reported RLS [9], potentially contributing to sleep difficulties, functional impairments, and lower quality of life. IRLS, which is a specific rating scale to RLS symptoms, may become increasingly used to indicate responsiveness to treatment. An ongoing study at Massachusetts General Hospital and University Medical Center Amsterdam uses the IRLS to assess the efficacy of pramipexole as a treatment for RLS in women with ALD [20].

#### 4.1.3 Study Objective Measures and Biomarkers That May Predict Longer‐Term Outcomes

Although the current paper only focused on clinical rating scales, objective measures assessing physiology or function may also be useful in outcomes assessment. Markers such as postural sway and gait are important quantitative assessments for men and women with ALD and should continue to be incorporated in future research. Keller and colleagues reported longitudinal analysis of quantitative sensorimotor data measured during 2005 to 2008 from 148 participants (61 men and 87 women) enrolled in a double‐blind placebo‐controlled trial to evaluate the effect of Lorenzo's Oil in adults with myeloneuropathy [21]. The analysis showed that postural sway amplitude measured on a force plate increased significantly over 2 years for men and women, a much shorter timeline of progression than clinical scales have detected. Hip flexion strength also decreased over 2 years for both sexes. More recently, Yska and colleagues identified that postural sway and gait speed measured by wearable accelerometers were strongly correlated with the EDSS score in a large, international cohort of women and men with ALD. Furthermore, women and men with ALD who required a walking aid to ambulate in the community showed a significantly higher degree of postural sway compared with patients who didn't use a walking aid [22].

Identification of imaging or blood biomarkers could also be useful as surrogate outcome measures that may be predictive of longer‐term outcomes [23, 24]. MRI techniques examining the white matter microstructure in the brain and spinal cord may be particularly beneficial in monitoring for subtle changes in disease pathophysiology [25]. Similarly, levels of C26:0‐lysophosphatidylcholine (C26:0‐lysoPC) in plasma and dried blood spots have emerged as superior biomarkers compared to classical VLCFA measurement, since C26:0‐lysoPC is elevated in more than 99% of affected women, including those whose VLCFA levels remain normal [26]. More recent studies have also shown that higher VLCFA‐lipid levels, including C26:0‐lysoPC, correlate with more severe disease manifestations in female ALD patients, suggesting utility for longitudinal monitoring and prognosis [27]. These markers may also respond more rapidly to intervention than functional or disability assessments, facilitating trials with fewer patients and shorter follow‐up. In conclusion, future natural history and treatment studies should consider both physiological and sensorimotor COAs, as well as those that capture treatment efficacy from the patient's perspective, through continued assessment of patient‐reported quality of life and perceived functional ability.

#### 4.1.4 Investigate the Efficacy of Pharmacological Therapies on Symptom Management

The effect of medication management on women with ALD has not been systematically studied. Some symptomatic women reported not being on chronic management such as drugs for spasticity or bladder dysfunctions [6]. One study collected current medications taken by the participants, but the information was not further utilized [7]. There is a need to perform medication reconciliation and assess how assessment results may differ based on whether individuals are on pharmacological treatments for specific symptoms or not.

#### 4.1.5 Investigate the Efficacy of Non‐Pharmacological Intervention

Non‐pharmacological interventions can help patients manage both physical health and mental health symptoms. As highlighted in the 2022 PFDD meeting, mobility challenges present significant quality of life challenges. One case report introduced therapeutic yoga to address movement and myelopathy symptoms [28]. Low‐risk, low‐cost physical therapy modalities are worth further exploration. Additionally, introduction to ALD support networks after diagnosis and psychosocial support surrounding various topics, including reproductive choices, parenting a child with ALD, and having uncertainty about the future, may better address the patients' social and emotional needs [29, 30, 31]. Integrating psychosocial support and making the group's voices heard are further steps toward learning about this population's experiences and raising public awareness of women with ALD. For example, a recent randomized‐controlled trial found that symptomatic women with ALD receiving regular online neurological, social, psychological, and nutritional counseling experienced improved quality of life after 6 months of participation compared to women in a waitlist control group who had not yet participated [32].

## 5 Conclusion

This scoping review provides an overview of clinical rating scales used in published studies, many of which were able to discriminate between asymptomatic and symptomatic women with ALD. Due to the slow progression rate and small, geographically dispersed patient populations, identifying factors contributing to disease progression in ALD is challenging. Outcome measures should not only be specific, reproducible, and sensitive longitudinally, but also be targeted to the specific symptoms the therapy is expected to address. The EDSS and IRLS may have adequate potential to differentiate between symptomatic and asymptomatic women, although they may only capture the slow, long‐term progression over a period of multiple years. Future studies could build momentum by further confirming the validity of these COAs, determining objective correlates that can be measured over shorter time scales, and focusing on the roles of both pharmacological and nonpharmacological interventions in supporting women with ALD.

## Author Contributions

Conceptualization of this article: Chenwei Yan, Reena V. Kartha. Design of this article: Chenwei Yan, Elizabeth I. Pierpont, Amena S. Fine, Reena V. Kartha. Writing, editing, and review of this article: Chenwei Yan, Elizabeth I. Pierpont, Amena S. Fine, Reena V. Kartha. Supervision: Reena V. Kartha, Elizabeth I. Pierpont.

## Funding

E.I.P. is supported by the National Center for Advancing Translational Sciences (UM1TR004405) and the National Institute for Neurological Disorders and Stroke (K23NS123258). The content is solely the responsibility of the authors and does not necessarily represent the official views of the National Institutes of Health.

## Ethics Statement

The authors have nothing to report.

## Consent

The authors have nothing to report.

## Conflicts of Interest

The authors declare no conflicts of interest.

## Supporting information


**Table S1:** Observational studies of women with ALD.

## Data Availability

Data sharing not applicable to this article as no datasets were generated or analyzed during the current study.
