# Supplementary material for: Clinically Relevant Outcome Measures in Women With Adrenoleukodystrophy
Source: Ann Clin Transl Neurol. 2026 Jan 20;13(4):646–53. doi: 10.1002/acn3.70314 (PMC13071096; doi:10.1002/acn3.70314)
Supplement: Supplementary file 1 — Table S1: Observational studies of women with ALD. [file ACN3-13-646-s001.docx]

**Supplementary Table 1.** Observational Studies of Women with ALD

| **Article (Author, year, country)** | **Study design** | **Setting** | **Population** | **Comparator: Symptomatic vs. Asymptomatic Women** |
| --- | --- | --- | --- | --- |
| Engelen M et al., 2014, Netherland | Cross-sectional study | Outpatient clinics of the Academic Medical Centre and the Medical Centre Alkmaar | Adult women (>18 years old) with ALD | Symptomatic defined as presence of myelopathy or peripheral neuropathy |
| Habekost CT et al., 2014, South Brazil | Cross-sectional study | Instituto Nacional de Genética Médica Populacional (INAGEMP), Porto Alegre, Brazil | All women (>18 years old) previously identified as heterozygotes for X-ALD in South Brazil and gave informed consent | Symptomatic defined as presence of neuropathic pain, paresthesia, sphincter dysfunction or paresis |
| Habekost CT et al., 2015, South Brazil | Prospective follow-up cohort of X-ALD of women whose cross-sectional findings have already been described (Habekost et al. 2014) | Instituto Nacional de Genética Médica Populacional, Porto Alegre, Brazil | All women (>18 years old) previously identified as heterozygotes for X-ALD in South Brazil | Symptomatic defined as presence of neuropathic pain, paresthesia, sphincter dysfunction or paresis |
| Schirinzi T et al., 2019, Italy | Single center, retrospective chart review (2010-2017) | Gesu Children's Hospital, Rome, Italy | All the genetically confirmed ABCD1 women, who were X-ALD men patients’ relatives, referred to the hospital | Symptomatic defined as having at least one of the following: AACS >1, tendon reflex responses (abnormal <1+, >3+), Babinski’s sign, muscle tone (abnormal >1 Ashworth scale). Asymptomatic group had a normal examination and no neurological complaints. |
| Huffnagel I et al., 2019, Netherland | Single center, cross-sectional study and follow-up on the group described in Engelen M et al. 2014 | Academic Medical Center, Amsterdam | Women with ALD reported by research group previously (Engelen M et al 2014) + newly identified women at the center | Symptomatic defined as presence of symptoms and signs of spinal cord disease |
| Schäfer L et al., 2023, international | Cross-sectional study | Questionnaires delivered either by mail or online via the web platform Leuconnect operated by ELA International | German-, English-, or French-speaking women aged ≥18 years | Symptomatic defined as reported signs of myelopathy and/or peripheral neuropathy in at least one domain |
